# Acute Presentation of Urachal Cyst: A Case Report

**DOI:** 10.7759/cureus.8220

**Published:** 2020-05-21

**Authors:** Sruthi Jayakumar, Danny Darlington

**Affiliations:** 1 Epidemiology and Public Health, Chest Research Foundation, Respiratory Research Network, Pune, IND; 2 Urology, Pondicherry Institute of Medical Sciences, Pondicherry, IND

**Keywords:** acute abdomen, sepsis, urachal cyst, urachus

## Abstract

Urachus is an embryonic organ related to the bladder that degenerates after birth. Defective obliteration of the urachus leads to urachal malformations, the most common of which is a urachal cyst. A urachal cyst is often misdiagnosed due to its myriad presentations. Delay in diagnosis can lead to complications such as sepsis, fistula formation, and rupture of the cyst mimicking peritonitis. Hence, a high index of suspicion is required for the timely diagnosis and management of urachal cysts presenting in the emergency room. We report the case of a 32-year-old woman who presented with clinical features suggestive of an acute abdomen. The judicious use of imaging confirmed the diagnosis of an infected urachal cyst, which was surgically managed.

## Introduction

Urachal cyst is an uncommon congenital anomaly that typically presents in older children. It very rarely presents in adults and the incidence is largely unknown in this age group. It arises from the incomplete obliteration of urachus, which is a primitive structure that connects the umbilical cord to the fetal bladder [1]. The incidence of urachal cyst is one in 5,000 live births [2]. It is mostly asymptomatic and around 35% of the patients present with lower abdominal pain, features of urinary tract infection, painful abdominal lump, and hematuria. We report the case of an acute presentation of a urachal cyst in an adult female patient that was diagnosed and managed successfully.

## Case presentation

A 32-year-old woman presented to the emergency department with acute onset of suprapubic pain of six-hour duration and low-grade fever of 38 °C. The pain was neither radiating nor migrating to other regions of the abdomen. She denied any history of trauma or comorbid illness. She had delivered two children through a lower segment cesarean section and had not undergone any other surgery. On examination, her vital signs were stable. Her abdomen was soft with a 5-cm sized tender swelling over the suprapubic region. A subcutaneous abscess was suspected based on the initial clinical findings.

**Figure 1:**
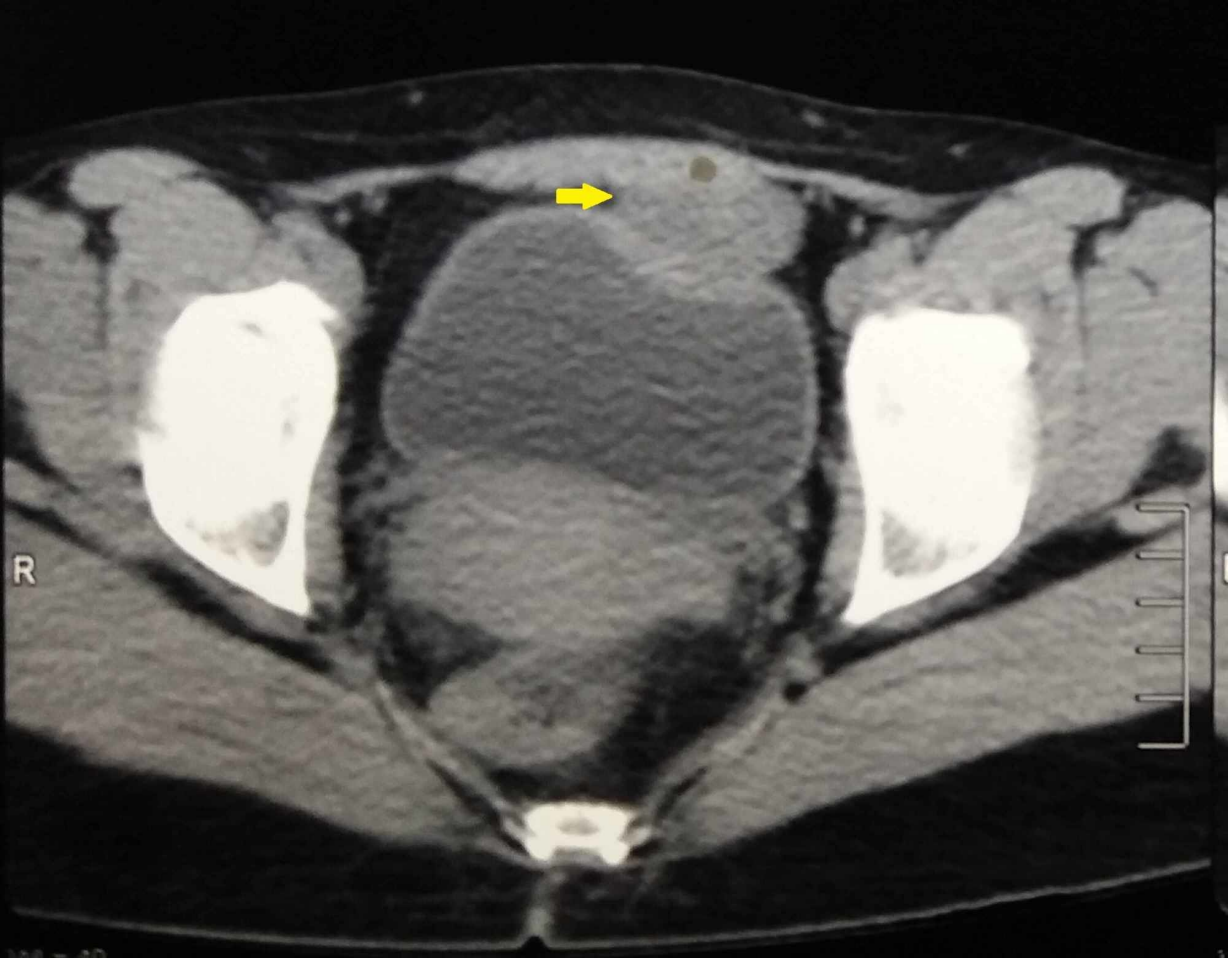
Transverse section of the CT showing the urachal cyst (arrow) anterior to the bladder CT: computed tomography

**Figure 2:**
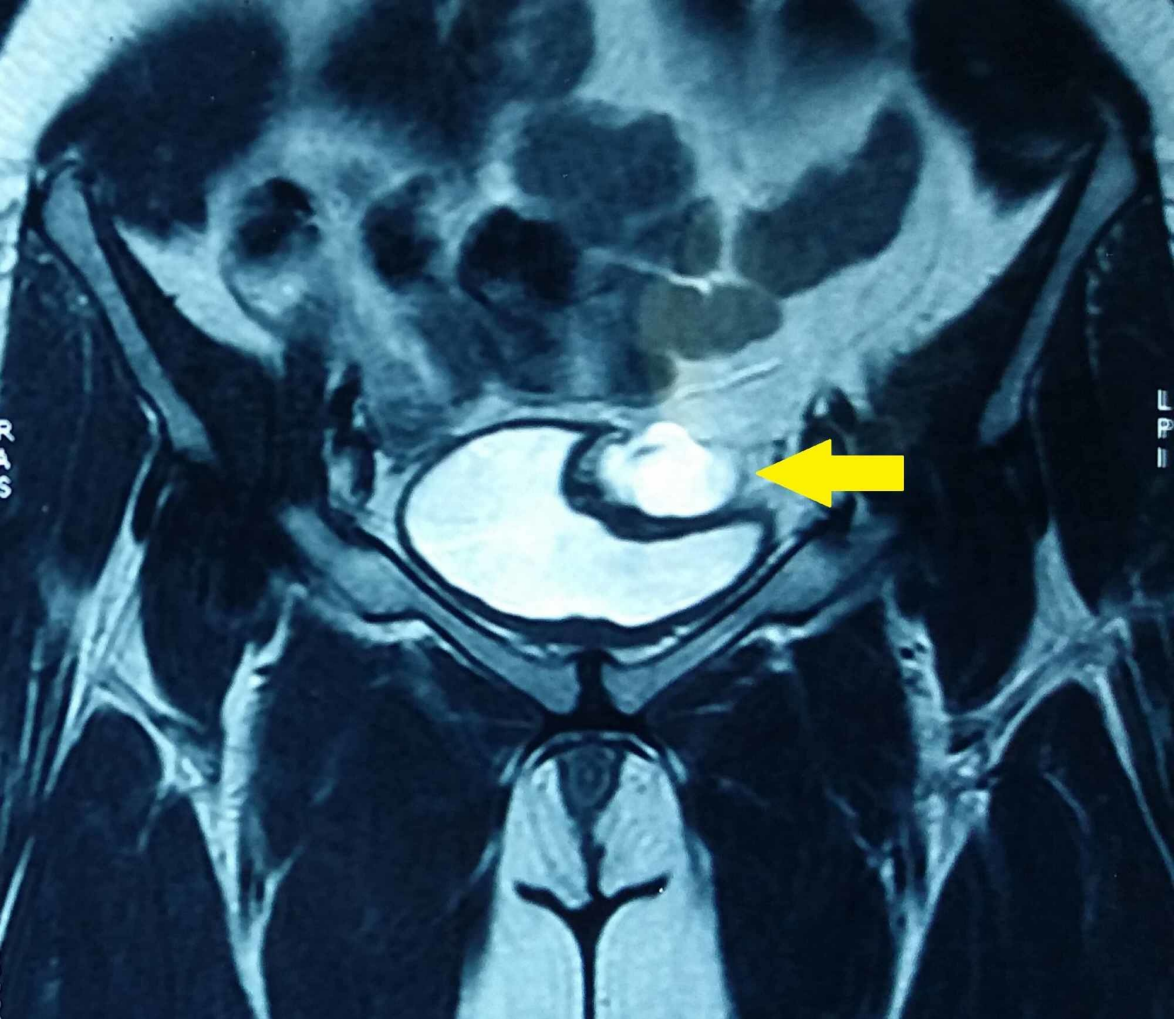
Coronal section of MRI depicting the hyperintense urachal cyst (arrow) in T2 sequence MRI: magnetic resonance imaging

The laboratory results including renal parameters, electrolytes, blood counts, and urine analysis were normal except for leucocytosis of 13,000 cells per ml (normal range: 4,000-12,000 cells per ml). Ultrasonography revealed a 5*5*5-cm sized hypoechoic lesion in the suprapubic region closely related to the dome of the bladder with internal echoes. CT of the abdomen and pelvis showed a 5*4*5-cm sized hypodense homogenous collection in the anterior abdominal wall close to the bladder (Figure 1). MRI revealed a hyperintense lesion on the T2 sequence occupying the prevesical space without communicating with the bladder (Figure 2). The imaging findings aided in narrowing down the preoperative diagnosis of a urachal cyst.

Given the above findings and the acute presentation, the patient underwent emergency exploration after informed consent. A transverse skin incision was made over the swelling and exploration was done, which revealed a 5-cm sized pus-filled cavity connected to the bladder dome by a fibrotic band. The abscess with its wall and the entire tract extending to skin and bladder were excised, and the skin was closed primarily after leaving a drain. Culture of the pus revealed *Escherichia coli* and appropriate antibiotics were started. Histological examination showed the cyst wall lined by stratified columnar epithelium, suggestive of a urachal cyst. The postoperative course was uneventful and the patient was discharged on the seventh postoperative day.

## Discussion

The urachus is a midline structure arising from the anterior aspect of the fetal bladder which in turn is derived from the ventral part of urogenital sinus [1]. During the later part of fetal life, the urachus progressively obliterates and is replaced by a fibrous tract in early adult life [2]. Failure of this obliteration can lead to anomalies of the urachus such as patent urachus, urachal sinus, and urachal cyst [2].

The urachal cyst is the most common urachal anomaly and is caused when both the proximal and distal portions of the urachus obliterate leaving a cystic cavity in the middle [3]. The incidence of urachal cyst in adults is unknown [4-5]. The cyst remains largely asymptomatic unless infected [6]. Infected urachal cysts present with acute symptoms such as suprapubic pain, dysuria, fever, nausea, vomiting, haematuria, pelvic pain, and purulent umbilical discharge [6-7]. The present case had suprapubic pain with low-grade fever at the time of admission. However the acute onset of pain caused diagnostic confusion, thereby delaying the correct diagnosis and treatment. Hence a high index of suspicion is needed to diagnose this extremely rare condition. In view of the diverse clinical presentation, the diagnosis of urachal cyst is often confused with obstructed hernia, appendicitis, Meckel diverticulitis, urinary tract infection, pelvic inflammatory disease, and bladder carcinoma [8].

Imaging techniques such as ultrasonography, CT, and MRI are the main modalities employed for the diagnosis of a urachal cyst [6]. The urachal cyst appears hypoechoic on ultrasonography and as a well-defined homogenous lesion occupying the prevesical space on CT [6]. Ultrasound can also reveal the presence of internal echoes within an infected cyst and can be used to guide diagnostic aspirations [9]. Microbiological study and culture of the abscess fluid are often done to rule out infection with *Escherichia coli*, *Enterococcus faecium*, and *Klebsiella pneumonia* being the common organisms isolated [7]. Imaging techniques provide information regarding the presence of urachal sinuses, the size of the cyst, and its relationship with the surrounding tissue [9]. The urachal cyst appears hyperintense on T2 weighted MRI sequence, which also delineates the anatomical relation of the cyst to the bladder [9]. Endoscopic evaluation by cystoscopy is rarely indicated when the imaging findings are ambiguous and cannot rule out communication with the bladder [10]. In the present case, CT and MRI have been used to confirm the diagnosis of the urachal cyst. The delay in diagnosis and treatment of the urachal cyst gives rise to various complications such as peritonitis, uracho-colonic fistula, stone formation, and neoplastic transformations [10-11].

The optimal management of the urachal cyst in adults is not standardized. Excision of the cyst can be done under antibiotic cover in one sitting or a two-staged procedure can be performed, which involves incision and drainage followed by delayed excision of the urachal remnant [12]. In the present case, incision and drainage of the abscess with complete excision of the cyst was performed in one sitting given the stable general condition of the patient. Some of the common complications of urachal cyst excision include wound infections, sepsis, fistula formation, and urinary leakage, and hence utmost care should be exercised to reduce the postoperative morbidity, especially in adults [13].

## Conclusions

Urachal anomalies are rare in adults and are often misdiagnosed due to the heterogeneous clinical presentation. Acute presentation of urachal cyst in adults is seldom encountered in clinical practice. Early diagnosis can help in planning appropriate surgical interventions, thereby reducing the morbidity. A high index of suspicion is of paramount importance for the timely diagnosis of this uncommon congenital anomaly.

## References

[1] Cappele O, Sibert L, Descargues J, Delmas V, Grise P (2001). A study of the anatomic features of the duct of the urachus. Surg Radiol Anat.

[2] Choi YJ, Kim JM, Ahn SY, Oh JT, Han SW, Lee JS (2006). Urachal anomalies in children: a single center experience. Yonsei Med J.

[3] Sherman JM, Rocker J, Rakovchik E (2015). Her belly button is leaking: a case of patent urachus. Pediatr Emerg Care.

[4] Qureshi K, Maskell D, McMillan C, Wijewardena C (2013). An infected urachal cyst presenting as an acute abdomen - a case report. Int J Surg Case Rep.

[5] Ekwueme KC, Parr NJ (2009). Infected urachal cyst in an adult: a case report and review of the literature. Cases J.

[6] Mrad DK, Ben Rhouma S, Zaghbib S, Oueslati A, Gharbi M, Nouira Y (2019). Infected urachal cyst in an adult: a case report. Urol Case Rep.

[7] Kaya S, Bacanakgıl BH, Soyman Z, Kerımova R, Battal Havare S, Kaya B (2015). An infected urachal cyst in an adult woman. Case Rep Obstet Gynecol.

[8] Ash A, Gujral R, Raio C (2012). Infected urachal cyst initially misdiagnosed as an incarcerated umbilical hernia. J Emerg Med.

[9] Yu JS, Kim KW, Lee HJ, Lee YJ, Yoon CS, Kim MJ (2001). Urachal remnant diseases: spectrum of CT and US findings. Radiographics.

[10] Mazzucchelli R, Scarpelli M, Montironi R (2003). Mucinous adenocarcinoma with superficial stromal invasion and villous adenoma of urachal remnants: a case report. J Clin Pathol.

[11] Venkat B, Kale S, Reddy SKBV, Govindaiah G, Mohammed IG, Panchal N (2017). "Look before you leap": urachal mass in adults. World J Oncol.

[12] Elkbuli A, Kinslow K, Ehrhardt JD, Hai S, McKenney M, Boneva D (2019). Surgical management for an infected urachal cyst in an adult: case report and literature review. Int J Surg Case Rep.

[13] Ashley RA, Inman BA, Routh JC, Rohlinger AL, Husmann DA, Kramer SA (2007). Urachal anomalies: a longitudinal study of urachal remnants in children and adults. J Urol.

